# Improving growth in preterm infants through nutrition: a practical overview

**DOI:** 10.3389/fnut.2024.1449022

**Published:** 2024-09-10

**Authors:** Domenico Umberto De Rose, Elena Maggiora, Giulia Maiocco, Daniela Morniroli, Giulia Vizzari, Valentina Tiraferri, Alessandra Coscia, Francesco Cresi, Andrea Dotta, Guglielmo Salvatori, Maria Lorella Giannì

**Affiliations:** ^1^Neonatal Intensive Care Unit, “Bambino Gesù” Children’s Hospital IRCCS, Rome, Italy; ^2^Faculty of Medicine and Surgery, “Tor Vergata” University of Rome, Rome, Italy; ^3^Neonatology Unit of the University, Department of Public Health and Pediatric Sciences, University of Turin, Turin, Italy; ^4^Neonatal Intensive Care Unit (NICU), Fondazione IRCCS Ca' Granda Ospedale Maggiore Policlinico, Milan, Italy; ^5^Dipartimento di Scienze Cliniche e di Comunità, Dipartimento di Eccellenza 2023–2027, University of Milan, Milan, Italy; ^6^Human Milk Bank, “Bambino Gesù” Children’s Hospital IRCCS, Rome, Italy

**Keywords:** extra-uterine growth restriction, parenteral nutrition, human milk, prematurity, fortification, fetal growth restriction

## Abstract

The primary purpose of this practical overview is to provide a practical update on appropriate nutritional strategies to improve growth in preterm infants. Current recommendations for improving preterm growth concern both macronutrients and micronutrients, with tailored nutrition since the first days of life, particularly when fetal growth restriction has been reported. Human milk is undoubtedly the best nutrition for all newborns, but, in some populations, if not adequately fortified, it does not adequately support their growth. In all preterms, growth should be correctly monitored weekly to intercept a negative trend of growth and implement nutritional strategies to avoid growth restriction. Similarly, growth should be accurately supported and monitored after discharge to improve long-term health consequences.

## 1 Introduction

Preterm infants have postnatal growth windows that are neurodevelopmentally sensitive both before and after the infant reaches term (1): increased energy and lipid intake in the early postnatal weeks has been linked to better neurodevelopmental outcomes, including a lower incidence of brain injury in magnetic resonance imaging (MRI) and a decreased level of brain dysmaturation at term equivalent age (with a more pronounced association for the gray matter) (2), and better developmental quotient scores (3). Better language developmental scores have been demonstrated in infants who received more proteins (4), but an excessive load of proteins may be harmful to neonatal metabolism and later neurodisability and has few short-term benefits for growth (5).

Indeed, an overly fast weight increase in term newborns is linked to a heightened risk of metabolic syndrome, a condition that is common in ex-preterm infants in later life (6).

The large observational EPICure study suggested that the key growth phase for metabolic health occurs much later in childhood (in preschool age) (7). Therefore, it can be hypothesized that promoting growth in early infancy could potentially eliminate the need for later catch-up growth, leading to improved neurodevelopmental outcomes without compromising future metabolic health (8).

The primary purpose of this practical overview is to provide an update on appropriate nutritional strategies to improve growth in preterm infants. We also provided some details about the tailored nutrition that some categories require, such as for the case of fetal growth restriction.

## 2 Methods

In order to create this narrative review, the terms “nutrition” and “neonate” were matched with “growth” in the PubMed database. All English-language papers that were recovered and released from 1990 to 30 June 2024 were analyzed without imposing restrictions on date, country, study design, outcomes, or inclusion/exclusion criteria. The reference lists of the identified papers were further checked, and each author identified additional references for this review based on their expertise in the relevant topic.

## 3 Macronutrients in the early postnatal phase (parenteral nutrition)

### 3.1 Glucose

Glucose is the primary energy source for the central nervous system and, thus, for its development. Preterm neonates have smaller hepatic glycogen stores at birth, and the proportion of glycogen concentration only rises after 36 weeks of gestation, making them more susceptible to developing hypoglycemia. Different thresholds for hypoglycemia have been proposed, but little is known about which one produces worse short-and long-term impacts (9, 10).

On the other hand, preterm infants are also often prone to hyperglycemia, which is associated with increased mortality and morbidity early in life due to the consequences of higher glucose levels in different organs (11).

The glucose infusion rate in preterm nutrition is most relevant in the critical early postnatal phase and in prescribing adequate parenteral nutrition. Once exclusive enteral nutrition is established, glucose intakes are generally more easily controlled by the infant’s own metabolic response provided there are no comorbidities.

Continuous glucose monitoring (CGM) offers a quantifiable measure of neonatal stress that reflects increased glucose variability during newborn care procedures (12). Utilizing CGM devices, combined with protocols for adjusting glucose infusion, might assist in keeping blood glucose levels within proposed ranges and decrease the frequency of blood sampling (13, 14).

The amount of glucose to be provided in preterm infants should start with 5.8–11.5 g/kg/day, considering the phase of illness and the supply of glucose and other macronutrients via parenteral and enteral nutrition. From day 2 onwards, glucose intake should gradually increase over 2–3 days up to 11.5–14.4 g/kg/day (15).

Excessive glucose intake should be avoided as it can lead to hyperglycemia (>180 mg/dL), which has been linked to increased morbidity and mortality (16).

### 3.2 Lipids

Lipids are a crucial energy source for premature infants and are vital for the intricate processes essential for normal central nervous system development (17).

A 25–50% lipid intake of non-protein calories is often advised in completely parenterally fed patients. Essential fatty acids (EFAs) are provided by lipids, which also aid in the transport of the lipid-soluble vitamins A, D, E, and K. Parenteral lipid intake in preterm newborns should not go beyond 4 g/kg/day. Intravenous lipid emulsions can be begun at the time of parenteral nutrition (PN) commencement and no later than day two following birth (18).

The delay in parenteral lipid introduction has been negatively related to weight gain up to day 28 of life and up to 36 weeks of postmenstrual age, independently from gestational age at birth, sex, and being small for gestational age (19).

Furthermore, early cumulative lipid intake in the first month of life has been associated with significantly greater cerebellar volume in a cohort of VLBW (Very Low Birth Weight, born weighing less than 1,500 g) infants on MRI obtained at term-equivalent age (20).

Newer multicomponent lipid emulsions (MLEs) in the last years, containing olive oil, fish oil and medium-chain triacylglycerols oil in addition to standard soybean oil, should be preferred to avoid administrating high levels of ω-6, which can increase proinflammatory eicosanoids biosynthesis leading to increased oxidative stress, and low levels of ω-3 as contained in pure soybean-based lipid emulsions (21, 22).

Indeed, higher ω-3 fatty acids docosahexaenoic acid (DHA) levels seemed to be associated with larger deep gray matter, cerebellar, and brainstem volumes at MRI studies performed at near-term age in a cohort of preterm neonates born <32 weeks GA. Kamino et al. reported that higher ω-3 and lower ω-6 fatty acid levels correlated with larger volumes: larger cortical and deep gray matter, cerebellar, and brainstem volumes by term age were associated with improved language scores and larger cerebellar and brainstem volumes with improved motor scores in pre-scholar age (30–36 months corrected age) (23).

MLEs might improve growth and neurological outcomes, delivering fats for brain growth that the traditional soybean-based lipid emulsions (SLEs) fail to provide. Vlaardingerbroek et al. demonstrated in VLBW infants that an MLE was well tolerated and associated with improved growth during the first 28 days of life compared to the pure SLE (24).

Then Costa et al. confirmed evidence from a randomized controlled trial that an MLE is associated with improved head circumference growth compared to a pure SLE from birth to 36 weeks PMA or at discharge (25).

### 3.3 Proteins

There is still debate over the ideal dose of amino acids to start with and the maximum amount that should be consumed because nutritional practices must prevent toxicity brought on by providing nutrients in excess of the preterm body’s ability to use them (26).

The estimated daily intake of amino acids for the fetus is 3 to 4 g per kilogram of body weight, which is considered to be sufficient for fetal growth and brain development (27).

Conversely, it is unknown how many proteins ELBW (Extremely Low Birth Weight, born weighing less than 1,000 g) infants should receive to support their growth and development, particularly in the first few days of life (28).

The amino acid supply should start on the first postnatal day with at least 1.5 g/kg/day to achieve an anabolic state, and then from postnatal day 2 forward, the parenteral amino acid intake should range from 2.5 g/kg/day to 3.5 g/kg/day and should be combined with non-protein intakes greater than 65 kcal/kg/day and sufficient vitamin intakes (29).

Indeed, almost 3.5 g/kg/body weight of amino acid is needed to retain 350 mg of nitrogen, enabling 17 g/kg/day growth (30).

The resting energy expenditure (REE) should also be considered: the majority of preterm infants have a greater REE (+140%), while full-term neonates exhibit lower needs (+47%). Furthermore, it is well known that comorbidities raise energy consumption, with consequent increased metabolic need, such as in the case of increased oxygen consumption of injured lungs (31).

Beyond observational studies reporting the association between higher levels of protein intake and better growth and neurodevelopment (32, 33), a double-blind, placebo-controlled trial (Protein Intravenous Nutrition on Development – ProVIDe trial) randomized neonates with birth weight lower than 1,000 grams to receive either amino acids at a dose of 1 gram per day (intervention group) or placebo in addition to usual nutrition for the first 5 days of life. The number of infants who survived without neurodevelopmental impairment at 2 years of life did not increase when additional parenteral amino acids were administered, but a higher relative risk of patent ductus arteriosus (RR 1.65; 95% CI 1.11–2.46) and refeeding syndrome (RR 1.64; 95% CI 1.09–2.47) were observed in this group (34).

## 4 Micronutrients in the early postnatal phase (parenteral nutrition)

### 4.1 Electrolyte supplementation

Electrolyte imbalances should be avoided since they may cause neurological morbidities and are related to growth failure (35, 36).

Refeeding syndrome (RS), which has been associated with high intravenous amino acid consumption in the setting of inadequate electrolyte supply (especially potassium and phosphorus), is widespread in ELBW infants, particularly in those who are small for gestational age (SGA), defined as a birth weight of less than the 10th percentile for gestational age (37–40). In this population, severe hypophosphatemia has been correlated to an elevated risk of intraventricular hemorrhage, and RS has been linked to a three-fold increase in death. Phosphate and calcium optimization in intravenous solutions may lessen RS and its effects (40). The incidence of hypophosphatemia in different cohorts of preterm infants ranges from 7 to 56%: it is difficult to compare the incidence across the different thresholds (<4.96 mg/dL, <4.65 mg/dL, <4.5 mg/dL, <4.3 mg/dL, or < 4 mg/dL) (41).

The balance of calcium and phosphorus requires simultaneous control of calcium and phosphorus intake (42). Lower calcium (Ca), phosphorus (P), and magnesium (Mg) intakes are advised for preterm newborns on early PN during their first few days of life than for developing stable preterm infants. Early PN should contain a molar Ca:P ratio below 1 (0.8–1.0) to lower the prevalence of early postnatal hypercalcemia and hypophosphatemia while protein and energy intakes are progressively increased (43).

Concerning sodium (Na), primary sodium depletion is common in preterm children born before 34 weeks GA due to insufficient proximal and distal tubule sodium reabsorption, which is worsened by pharmacological side effects from substances like diuretics (44).

The major sources of salt for the preterm neonate include the prescribed PN, blood product transfusions, flush solutions, and saline infusions. Supplementing parenteral feeding with sodium glycerophosphate since the first day of birth considerably reduced the incidence of hypophosphatemia, according to Bustos Lozano’s findings, who compared a prospective cohort study of infants ≤1,250 g receiving sodium glycerophosphate from the first day of life and a previous subcohort supplied with phosphate after 48 h (45, 46).

Hyponatremia (<135 mEq/L) is not as benign as previously thought since sodium is an essential ingredient found in bone, cartilage, and connective tissue; it is also required for the correct development of the central nervous system (47). Sodium supplementation could have a positive impact on weight gain (36, 48).

The amount of 1–3 mmol of sodium and 1–3 mmol of potassium required per intake of 100 kcal for newborns and children beyond the neonatal stage is mostly based on empirical findings (44). In addition to the risks of metabolic acidosis and hyperchloremia, supplementing sodium losses only with sodium chloride solutions could expose patients to excessive cumulative chloride consumption (35).

Sodium lactate or sodium acetate-based solutions, rather than sodium chloride solutions, could prevent these undesirable metabolic effects (49).

Moreover, potassium (K) intake should coincide with the delivery of amino acids to prevent refeeding syndrome. As a result, enough K intake is also necessary when supplying early high amino acids and energy with early PN (44).

On the other hand, it can be crucial that sizeable quantities of sodium and potassium be provided with medications (such as antibiotics) and minerals that are prepared as Na or K salts (e.g., phosphates) (44).

Considering SGA infants, potassium and phosphorus intakes should be set at sufficient levels from birth onwards because they have a high risk of early hypokalemia and hypophosphatemia (38).

### 4.2 Vitamins

A sufficient vitamin intake is essential for correct growth and development. Among fat-soluble vitamins, vitamin A plays an essential role in growth: preterm infants on PN should receive 700–1500 IU/kg/day of vitamin A, and VLBW neonates often suffer from vitamin A deficiency. Considering that there are significant losses of vitamin A when administered with a water-soluble solution, parenteral lipid-soluble vitamins should always be administered with a lipid emulsion when possible (50).

Furthermore, vitamin A deficiency has been related to a higher risk for bronchopulmonary dysplasia (BPD) and retinopathy of prematurity (ROP) (51, 52).

Vitamin D has a role in growth because it increases intestinal absorption of calcium and phosphorus and enhances bone mineralization. The available research, as well as most guidelines, recommend an intake of 400 IU daily of vitamin D as adequate for bone health in preterm and full-term infants (53), whereas in Europe, according to the European Society for Pediatric Gastroenterology, Hepatology, and Nutrition (ESPGHAN) guidelines, the authors suggest a higher supplementation of vitamin D for preterm infants, reaching up to 800–1,000 IU/day, to rapidly correct the fetal low plasma levels of vitamin D (54). Enteral vitamin D supplementation in preterm or LBW infants allows an increase in weight-for-age z-scores and height-for-age z-scores at 6 months, according to a recent meta-analysis about infants who received vitamin D (200 IU to 800 IU per day). However, there were no effects on neurodevelopmental outcomes in the vitamin D supplementation groups (55).

Among water-soluble vitamins, folic acid (FA, also known as vitamin B9) has a crucial role in promoting fast cell division and proliferation, which is crucial for the growth and development of the fetus and, accordingly, of the preterm neonate. Preterm infants should receive 56 mcg/kg/day of folic acid (50).

Indeed, in the first two to three months of life, preterm neonates have low blood FA levels: rapid growth, an increase in erythropoiesis, the use of antibiotics or anticonvulsants, and intestinal immaturity with malabsorption are some of the factors that may have an impact on the hepatic FA reserves, potentially resulting in insufficiency (56, 57).

Maternal supplementation during pregnancy and smoking are the two most significant variables affecting blood FA levels during the first month of life, considering that smoking exposure during pregnancy is generally associated with lower folate levels (58, 59). Women who smoked cigarettes during pregnancy and took higher-dose folic acid supplements delivered infants with better fetal growth than smokers who took standard-dose folic acid (60).

### 4.3 Minerals: zinc and iron

Zinc (Zn) is a mineral essential for growth due to the increased enzyme activity and synthesis of proteins for tissues: clinical manifestations of its deficiency are growth failure, increased infection rate, and a characteristic skin rash (61).

Zinc should be provided with PN at a 450–500 mcg/kg/day dose in preterm infants to match *in-utero* growth rate. Standard trace element preparations do not supply this amount, and additional Zn has to be added to PN fluid in the preterm infant or those patients with high Zn losses (e.g., from diarrhea, stoma losses, or severe skin disease) (62, 63).

Furthermore, zinc supplementation for more than 2 weeks seems to improve head circumference growth (64), and this preliminary result has also been confirmed by a positive correlation between zinc levels at 28 days of life and next motor scores at 12 months of life (65).

Any preterm infant who is not developing normally despite receiving an apparently adequate amount of energy and macronutrients, who has not consistently consumed zinc-fortified human milk or formula made specifically for preterm infants, who underwent any gastrointestinal resection or injury, or who is receiving diuretic therapy, should be investigated for zinc deficiency (66).

LBW infants are also at high risk of iron deficiency due to low iron stores at birth and higher iron requirements due to rapid growth (67). Enteral iron supplementation in these infants increases linear growth (length) but has little effect on weight, head circumference, or cognitive development, according to a meta-analysis including eight trials. However, the anthropometric tools used in the different papers vary greatly: thus, it is important to proceed with care when evaluating these results (68).

## 5 Enteral nutrition

Early enteral feeding can prevent villous atrophy, reduce intestinal permeability, and improve the microbiota (69–71). Observational studies showed that standardizing enteral feeding practices in the Neonatal Intensive Care Unit (NICU) improves preterm infants’ ability to receive adequate enteral feedings. Unit recommendations allow for short PN duration and hospital stays to lower the incidence of necrotizing enterocolitis (NEC) and maybe improve neurological development (72).

Recent research indicates that enteral feedings can be pushed by up to 20–30 mL/kg/day (where clinically acceptable) in the days following delivery (72–74). Early removal of peripheral and central catheters reduces discomfort, infection risk, and PN-related problems in newborns (75, 76).

## 6 Human milk, fortification, and growth

Human milk is recommended as the natural feeding for preterm infants and as a cost-efficacy strategy for lowering morbidity and economic burdens. Although related to a slower weight gain than formula feeding, human milk is associated with better recovery of body composition through fat-free mass deposition, which may eventually result in superior metabolic and neurodevelopmental outcomes (77, 78).

To optimize growth with human milk in general (and donor milk specifically), an adequate fortification of human milk is recommended to avoid negative trends in weight Z-scores in VLBW infants (79). Unfortified human milk at usual feeding volumes (160 mL/kg/day) does not meet the recommended energy provision (110–130 kcal/kg/day) or enteral protein intake of 3.5–4.5 g/kg/day for VLBW infants (80).

Furthermore, pasteurization and milk banking processes can reduce donor milk’s fat and protein content (81).

Fortification is generally meant using multi-nutrient fortifiers rather than single-nutrient fortifiers. When adopting individualized strategies of fortification, single-nutrient fortifiers are useful for adjusting the amount of protein, carbohydrates, lipids, and calories (82).

An example of how to use a combination of multi-nutrient and single-nutrient fortifiers in a regimen of adjustable fortification is reported in Table 1.

**Table 1:** A possible method to adjust the fortification of HM according to blood urea nitrogen (BUN) values is to assess it at least once weekly.

	Level of fortification according to BUN levels	
BUN level	Multi-nutrient fortifier	Only-protein fortifier
<10 mg/dL	4%	0.4% (If low BUN levels persist, increase up to 0.8% and then up to 1.2%)
10–25 mg/dL	4%	–
>25 mg/dL	2% (If high BUN levels persist, reduce to 1%)	–

Then, different types of fortifiers are available: bovine or donkey milk-based fortifiers or human milk-derived fortifiers with powder or liquid formulations, but there is still no consensus about the best option (80, 83).

A recent randomized controlled trial demonstrated no clinically relevant differences in body composition (adipose tissue and non-adipose tissue mass) at whole-body magnetic resonance imaging at term age in preterm babies <30 weeks GA who received a macronutrient-equivalent exclusive human milk diet (human milk formula in case of lack of own mother’s milk and human milk derived fortifier) compared with a diet containing cow milk products (preterm formula in case of lack of own mother’s milk and cow milk-based fortifier) (84).

However, a recent meta-analysis suggests that an exclusive human milk diet could possibly be associated with decreased mortality with no association with major complications (NEC, BPD, sepsis, and ROP) (85).

Along with deciding the type of fortifier to use, it may be difficult and is up for controversy in the NICUs when to add fortifier to human milk (72). Considering the limited published data on this topic, whether early introduction of fortification at an enteral feed volume of ≤40 mL versus delayed at ≥75 mL/kg/day improves growth or influences adverse feeding outcomes is still reason for debate (86).

Recent studies promote a move toward an earlier fortification (30 mL/Kg/day) to obtain a faster regain of birth weight and higher length and head circumference velocity without increasing the risk of feeding intolerance and NEC (87).

Evidence of the safety of early fortification, even when started at the first feed, is emerging (88).

**Figure 1:**
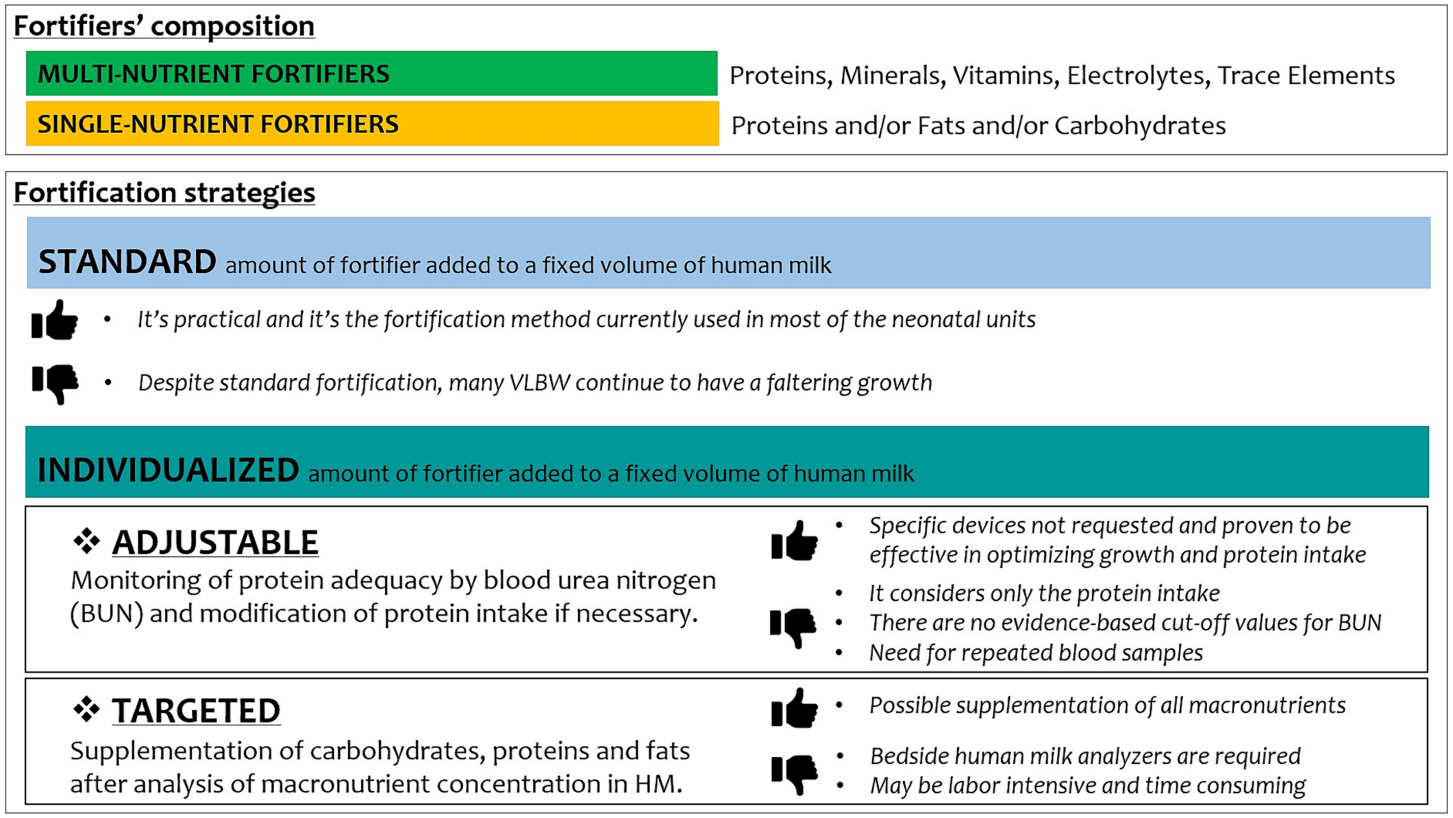
Fortifiers’ composition and human milk fortification strategies.

Among fortification strategies, adjustable (based on BUN values) or targeted (based on human milk analysis) increased growth velocity of weight, length, and head circumference as compared to standard fortification (Figure 1) (89).

A tailored fortification of human milk could result in higher weekly weight gain and daily growth rates, rather than a standard approach, as reported by Morlacchi et al., with osmolality values after target fortification remaining within recommended limits (90).

## 7 Tailored nutrition and growth in different subcategories

It is well known that within the preterm population, several categories are distinctive in their needs and nutritional risk. Among them, VLBWs, and even more so ELBWs, or premature infants with a history of fetal growth restriction (FGR), are at risk of impaired growth and need tailored nutritional interventions (33). This specific consideration is critical both in the early stages of parenteral nutrition but becomes even more so in the often-difficult transition between parenteral and enteral and then in exclusive enteral nutrition.

Most guidelines on pediatric parenteral nutrition concluded that standard PN solutions should be utilized rather than individualized PN solutions in most neonates, including VLBW ones, according to current studies. An individually tailored PN solution should be used when the nutritional needs cannot be met by standard PN formulations (i.e., critically ill and metabolically unstable patients, such as those with abnormal fluid and electrolyte losses, and infants needing PN for extended periods, such as those with short bowel syndrome) (91).

Tailored nutrition should consider that the fetus exhibits sex-specific growth differences relatively early during pregnancy. Therefore, considering that boys born preterm are recognized to be at higher risk of adverse outcomes than girls born preterm (92), the nutritional needs of males and females should also be different. However, there are few available studies about sex differences in neonatal outcomes, neonatal body composition and metabolism, and nutritional interventions (93–96).

Human milk composition studies confirmed the possible sex differences in nutritional requirements: (a) higher calories, carbohydrates, and fats provided to males than females; (b) same protein levels; (c) higher glutamine, glycine, cysteine, and tyrosine levels in males; and (d) higher taurine levels in females. From this point of view, the mother’s own milk (MOM) would give sex-specific development benefits, which are most likely due to the calibration of a mother’s milk depending on her newborn’s sex. Formula composition does not vary with baby gender, which might explain why body composition data promotes the use of OMM over formula (97).

Based on the observational outcome results following a randomized controlled trial of early aminoacid administration in preterm infants, van den Akker et al. reported that boys had a normal outcome significantly more often if aminoacids were administered from birth onward (OR 6.2, 95% CI 1.0–38.0) (93). Conversely, Alur et al. hypothesized that female ELBW infants may need higher protein and calories than male ones and failed to obtain different discharge outcomes between the two sexes despite differences in protein and calorie provision (98). Larger prospective studies of neonatal nutritional interventions are needed to focus on these aspects and should consider infant sex (96).

Furthermore, some SGA preterm newborns have a history of fetal growth restriction (FGR), a condition where the growth of the fetus during pregnancy is lower than expected based on the race, gestational age, and sex of the baby. This is thought to be the result of persistent malnutrition throughout the fetal period and is caused by placental dysfunction. Therefore, the refeeding syndrome may be to blame for the electrolyte imbalance seen in these newborns. In VLBW infants, the umbilical artery resistance index may be a helpful indicator of the potential emergence of hypophosphatemia resembling the refeeding syndrome. In these cases, a more aggressive nutritional strategy should be avoided; moreover, close monitoring of blood phosphorus and potassium levels, as well as early intervention, is mandatory (99).

## 8 The role of fetal growth restriction

**Figure 2:**
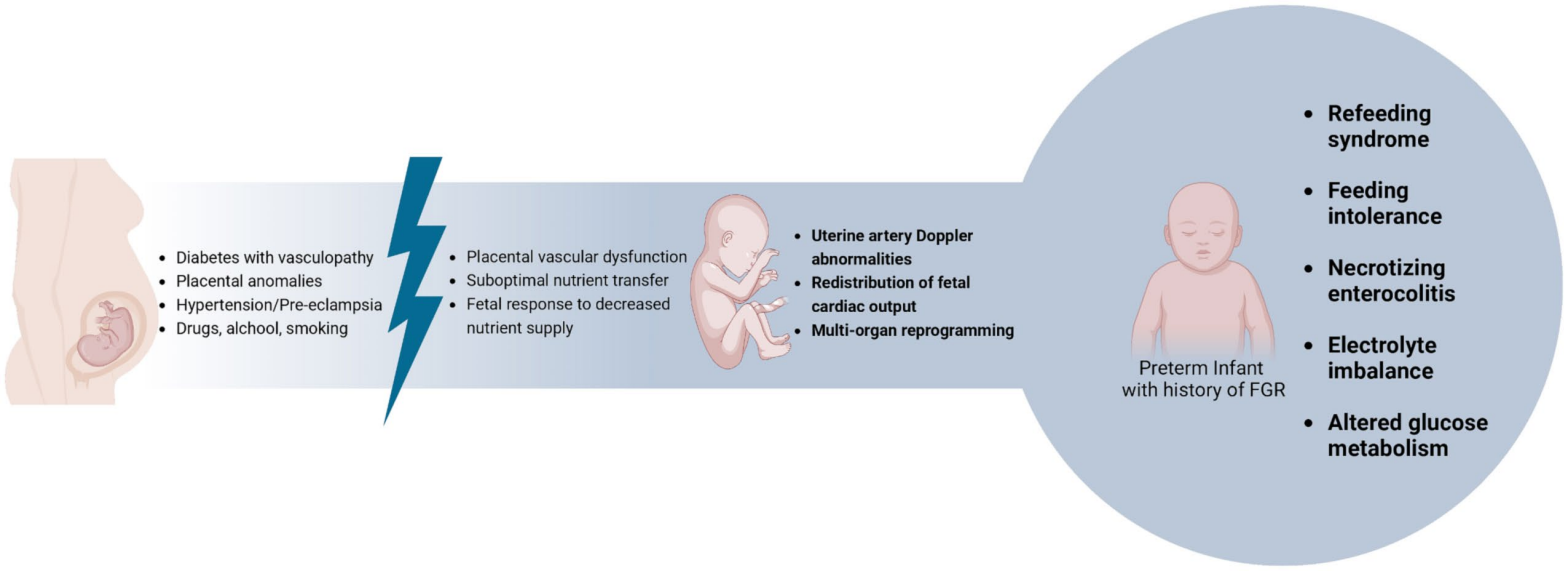
Fetal growth restriction and related risks for preterm infants.

Fetal Growth Restriction (FGR) may arise due to dysregulation or imbalance in the complex systems that regulate fetal growth (Figure 2) (100).

Anthropometric measurements and functional parameters, such as Doppler abnormalities, are used to distinguish newborns with FGR from those who are constitutionally SGA. However, studies suggest that infants with birth weight below the 3rd percentile compared to reference curves are at an increased risk of adverse short-and long-term outcomes, whether there were functional abnormalities during pregnancy (100, 101).

The fetus exposed to intrauterine growth restriction undergoes multi-organ reprogramming due to low placental blood flow. This results in the redistribution of fetal cardiac output to vital organs, leading to various postnatal complications, including gastrointestinal ones (102–104).

Numerous studies have shown that newborns with FGR are at greater risk of experiencing poor food tolerance (104), necrotizing enterocolitis (105), altered glucose metabolism (106, 107), and electrolyte anomalies after birth (108). Currently, there are no established guidelines to assist us in managing the nutrition of newborns with FGR. However, given the extremely delicate nature of these infants, it is crucial to provide appropriate and timely care after birth to prevent and treat any perinatal complications using effective nutritional strategies (102).

There has been much discussion regarding the right timing for introducing enteral nutrition. Tewari et al. conducted a randomized study on a group of premature infants with gestational age below 32 weeks who showed Doppler evidence of FGR. The study concluded that newborns who were fed early (within the first 2 days after birth) achieved full enteral feeding more quickly than newborns fed later (5–6 days after birth or later). Additionally, there were no significant adverse effects such as NEC (109).

Similarly, Aradhya et al. conducted a retrospective study that showed that starting early enteral feeding within 24 h of life is safe if there are no warning signs or symptoms. This practice is also linked with achieving full enteral feeding at a median age of 9 days (110).

It is crucial to assess both the timing of the start of enteral nutrition and the speed of daily increment to achieve full enteral feeding and evaluate food tolerance. In a large randomized controlled trial conducted by Dorling et al., premature infants weighing less than 1,500 g were divided into two groups: rapid increases (30 mL/kg/day) of enteral nutrition and slow increases (18 mL/kg/day). The two groups were compared, and no significant differences were observed regarding survival, survival without neurodevelopmental disability, and incidence of NEC (111). It is crucial to highlight that 90% of the newborns enrolled in the study were fed with any human milk. Among the infants who were formula-fed, only 40% of babies in the rapid-rising group survived without neurodevelopmental disabilities, compared to 70% in the slow-rising group (111).

Interestingly, Dani et al. analyzed the effects of human milk (HM), fortified HM, and formula milk on splanchnic oxygenation in preterm infants. Mother’s own milk feeding did not impair the gut tissue oxygen delivery extraction balance, and this might represent a protective mechanism against the development of hypoxic–ischemic injuries (112). Previously, Grometto et al. also studied near-infrared spectroscopy for monitoring the effects of feeding regimens on the cerebral and splanchnic regions: they found that gut oxygenation did not change during preterm formula feeding and increased after it (113).

Moreover, a research study conducted by Surmeli Onay et al. examined splanchnic oxygenation in a group of prematurely born infants affected by FGR. The aim was to determine if there were any significant differences in feeding tolerance between continuous feeding and intermittent feeding (bolus in 10 min). The study concluded that being fed by enteral route, rather than the feeding method, was the critical factor affecting splanchnic oxygenation and, therefore, feeding tolerance (114).

## 9 Tailored nutrition and extra-uterine growth restriction

The parenteral to enteral transition is a crucial phase for preterm infants, because up to 46% incidence of poor growth occurs during this period (115), and thus it’s important to individualize nutrition based on the needs and growth trajectories of the patient (Table 2).

**Table 2:** Current recommendations for improving preterm growth.

Macronutrients			
Glucose	Start with 5.8–11.5 g/kg/day (15)	Hyperglycaemia (>145 mg/dL) is associated with increased morbidity and mortality (16)	Continuous glucose monitoring (CGM) might assist in keeping blood glucose levels within the proposed ranges (13, 14)
Lipids	A 25–50% lipid intake of non-protein calories is often advised (18)	Parenteral lipid intake in preterm newborns should not go beyond 4 g/kg/day (18)	Newer multicomponent lipid emulsions (MLEs) should be preferred (21)
Proteins	Start with at least 1.5 g/kg/day to achieve an anabolic state (29)	From day 2, intake should range from 2.5 g/kg/day to 3.5 g/kg/day (29)	Protein intake should be combined with non-protein energy intakes greater than 65 kcal/kg/day (29)

Micronutrients			
Electrolyte supplementation	Lower Ca, P, and Mg intakes are advised during PN’s first few days (43)	Primary Na depletion is common in preterm children born before 34 weeks GA (44)	SGA infants have a high risk of early hypokalaemia and hypophosphatemia (38)
Vitamins	Infants on PN should receive 700–1500 IU/kg/day of vitamin A (54)	Enteral vitamin D supplementation in preterm allows an increase in weight and height at 6 months (55)	Folic acid has a crucial role in the preterm neonate. Preterm infants should receive 56 mcg/kg/day of folic acid (50)
Minerals	Zinc should be provided with PN at a 450–500 mcg/kg/day dose in preterm infants to match the *in-utero* growth rate (62)	Zinc supplementation for more than 2 weeks seems to improve head circumference growth (64)	Enteral iron supplementation in LBW increases length but has little effect on weight, head circumference, or cognitive development (68)

Tailored nutrition			
Fetal growth restriction	In VLBW infants, a history of FGR may be linked to the emergence of hypophosphatemia (38)	Starting early enteral feeding within 24 h of life is safe if there are no warning signs or symptoms (110)	Feeding tolerance and protective effects against comorbidities were associated with mother’s milk and not with formula (97)
Human milk fortification	Adequate fortification of human milk is necessary for VLBW infants (79)	Adjustable or targeted fortification strategies increase growth velocity compared to standard fortification (82)	Recent studies promote a move toward an earlier fortification (30 mL/Kg/day) to obtain an improved growth velocity (87)
Extra-uterine growth restriction	Parenteral to enteral transition is crucial: up to 46% incidence of poor growth occurs during this period (115)	Monitoring preterm infants’ growth during the hospital stay is crucial to detect early EUGR (118, 120, 121)	Measurements should be plotted on a growth chart derived from a growth standard appropriate to the population in question rather than the standard birthweight-derived charts (8)

Extra-uterine growth restriction (EUGR) is a term commonly utilized to describe poor growth in these infants, but there is no consensus on the type of definition to use. Two types of definitions are mainly available in the literature:

1. “Cross-sectional” definitions, that identify a cut-off below which the neonate is labeled as EUGR at a specific time-point (traditionally, as weight below the 10th percentile at discharge) (116);
2. “Longitudinal” definitions that describe the growth trends, reporting the differences between two-time points, usually from birth (i.e., weight loss more than one or two standard deviations from that at birth) (117, 118).

Recently, a new approach of “true EUGR” has been proposed, considering EUGR always cross-sectionally or longitudinally, but including only infants with appropriate age weight (119).

We believe that it’s crucial to have strict monitoring of preterm infants’ growth during the hospital stay, calculating Z-scores in weight and head circumference from when physiological weight loss is over to identify EUGR early and improve outcomes, according to different studies (118, 120, 121).

Measurements should be plotted on a growth chart derived from a growth standard appropriate to the population in question rather than the standard birthweight-derived charts. A standardized approach to providing enteral and parenteral nutrition has been shown to improve growth (8).

## 10 Possible causes of inadequate growth and strategies

The cause of growth restriction in preterm infants is multifactorial, but it has been postulated that nutrition accounts for around 50% of the variation in early postnatal growth, and the real amount of milk provided and tolerated plays a key role (122).

Furthermore, we do not know well the growth target we want to reach and that the presence of other comorbidities must be taken into consideration.

Independent risk factors of EUGR are moderate-to-severe bronchopulmonary dysplasia (BPD), gestational hypertension, cesarean section, cumulative fasting time, time required to achieve an adequate amount of enteral feeding, and hemodynamically significant patent ductus arteriosus (hsPDA) (123). In particular, infants with BPD seemed to show an unsteady pattern of growth compromise during the NICU stay (124).

Among minor factors associated with failure to thrive in preterm infants, gastroesophageal reflux (GER) is a common finding, with difficulty in differentiating between physiologic GER and GER disease (GERD) (125–127). A simple positioning approach can sometimes solve the problem, whereas there is little empirical evidence to support using feed thickeners in this age category (128, 129). Although a clear link between thickened feeds and undesirable gastrointestinal effects been not still found (130, 131), there is growing clinical concern regarding the use of thickened fluids in preterm infants and the development of NEC (132–134).

When these first measures (thickened formulas and physical approaches) are ineffective, Multichannel Intraluminal Impedance and pH Monitoring (MII/pH) with symptom correlation approaches may be necessary (125, 126). In the case of acid GER, alginates can represent a physical barrier to reducing esophageal acid exposure frequency (135, 136).

When preterm infants reach term or are beyond the full term, a cautious trial of acid suppression may be considered, carefully assessing the advantages over the potential risks (constipation, osteopenia, nausea, fatigue, abdominal pain, small intestinal bacterial overgrowth, necrotizing enterocolitis) (127, 137). Empiric treatment should not be used when arching/irritability is present, considering that extreme prematurity and neurologic impairment may more likely cause the arching/irritability (138).

Sometimes, factors associated with faltering growth are behavioral, such as poor milk drinkers, fussy eaters, and infants with oral aversion (139).

When the amount of milk administered seems to be adequate, other medical conditions should be ruled out (e.g., heart disorders, cystic fibrosis, malabsorption, or metabolic anomalies) (139). The impact of chronic conditions such as BPD and hsPDA should be weighed, considering the impact of administering large amounts of liquids to provide adequate nutrients in these infants (44, 140, 141).

If inadequate growth persists, once the term is reached, high-energy isocaloric infant formulas are widely used to provide adequate nutrients, restricting the fluids supplied and combatting poor growth. These high-energy feeds (1 kcaL/mL) are not designed specifically for formerly preterm infants, and the literature lacks clear evidence about their use in this age category. When feeding intolerance also coexists (defined as the inability to digest enteral feedings associated with increased gastric residuals, abdominal distension, vomits and/or regurgitations, abnormal feces, and associated cardiorespiratory events) (142, 143), an extensively hydrolyzed protein high-energy isocaloric infant formula could be indicated, although the lack of evidence. These formulas could be useful because they enhance energy and protein levels without mixing the milk with further products and reduce the risk of bacterial contamination and preparation errors.

## 11 Anthropometric follow-up of preterm infants

Early detection of post-natal growth retardation is essential in order to act promptly by fortifying human milk, adjusting the nutrient composition and caloric intake, or changing feeding methods. Anyway, preterm infants have a significant risk of metabolic disease due to their intrinsic vulnerability: they have a different body composition than their term peers, and monitoring body composition changes appears to predict long-term effects on health outcomes (117). We can intervene with our nutritional strategies, but this is not the only factor in programming long-term health outcomes. Excessive weight gain in the first months is associated with an increase in fat mass rather than in lean mass (144, 145) and this should be avoided to lower the risk of facing cardiovascular and metabolic diseases in adult life (146, 147). On the other hand, growth retardation can have significant long-term health consequences, such as an increased risk of chronic health issues (148, 149).

Therefore, surveillance of growth patterns is strongly recommended, even after NICU discharge. The most accurate and precise way to monitor infants’ growth is by measuring the three main anthropometric measurements: weight, length, and head circumference. When monitoring post-natal growth, it is important to use an appropriate growth chart, like the ones indicated for preterm infants, the INTERGROWTH-21st (150).

Preterm infants, especially the ones who face IUGR, are usually born SGA. At least 85–90% of children born SGA experience catch-up growth in the first 12–24 months of life. The remaining 10–15%, who do not reach a height over-2 SDS at the age of 2 years old, have a greater risk of remaining short as an adult (151–153).

If changing feeding strategies is not enough to guarantee optimal growth, it is possible to intervene pharmacologically. Short children born SGA who fail to demonstrate catch-up growth are candidates for growth hormone (GH) treatment. The Food and Drug Administration (FDA) and the European Medicines Agency (EMA), in 2001 and 2003, respectively, approved GH treatment for infants born SGA. The FDA approves the use of GH (0,067 mg/kg/die) in all SGA infants who fail to reach catch-up growth by the age of two. The EMA approves the use of GH (0,033 mg/kg/die) in infants born SGA that at the age of four still have a height < -2 SDS or a growth velocity < 0 and there is a distance to a mid-parental height of – 1 SDS (154, 155).

GH treatment is effective and safe. Its efficacy has been reassured by different studies: long-term GH treatment in short children born SGA leads to a normalization of adult height, even with a dose of 0.033 mg/kg/die (156, 157).

Even if, over the years, there were doubts about the increased risk of cancer, mortality, and cardiovascular disease caused by GH treatment, multiple studies have confirmed that, for approved indications and at recommended doses, GH treatment is safe to use and has no long-term negative consequences on health (158, 159).

The younger the patient is at the beginning of treatment, the more favorable the response. Hence, the importance of monitoring growth in preterm infants, especially the ones born SGA (155).

## 12 Conclusion

The postnatal growth window is crucial to ensure adequate outcomes for extremely preterm infants. As this review has emphasized, right from parenteral nutrition, it is necessary to consider the characteristics of the single infant and provide adequate nutrients (glucose, lipids, and proteins) and micronutrients (electrolytes, vitamins, and minerals) both quantity and quality-wise. Early start of enteral feeding within 24 h of life is safe, if there are no warning signs or symptoms and can allow to reach earlier full enteral feeding, advancing meals by up to 20–30 mL/kg every day (where clinically acceptable). This is related to a lower duration of the parenteral nutrition, and an earlier removal of central venous catheter, with the reduction of related complications. With the establishment of full enteral nutrition, hopefully with human milk, adequate fortification of human milk is necessary: adjustable or targeted fortification strategies increase growth velocity compared to standard fortification. The monitoring of the trend of growth parameters (weight, length, and head circumference), assessed once a week during hospitalization, can allow for a decrease in extra-uterine growth restriction by tailoring the newborn’s nutrition and leading to better outcomes. Finally, it is important not to forget the importance of adequate growth even after discharge, as poor growth is associated with long-term adverse outcomes.
